# UWB indoor positioning optimization algorithm based on genetic annealing and clustering analysis

**DOI:** 10.3389/fnbot.2022.715440

**Published:** 2022-07-26

**Authors:** Hua Guo, Mengqi Li, Xuejing Zhang, Xiaotian Gao, Qian Liu

**Affiliations:** College of Electronic and Information Engineering, Shandong University of Science and Technology, Qingdao, China

**Keywords:** UWB, triangulation method, fuzzy c-means, annealing evolution algorithm, positioning

## Abstract

Indoor location information is an indispensable parameter for modern intelligent warehouse management and robot navigation. Indoor wireless positioning exhibits large errors due to factors such as indoor non-line-of-sight (NLOS) obstructions. In the present study, the error value under the time of arrival (TOA) algorithm was evaluated, and the trilateral positioning method was optimized to minimize the errors. An optimization algorithm for indoor ultra-wideband (UWB) positioning was designed, which was referred as annealing evolution and clustering fusion optimization algorithm. The algorithm exploited the good local search capability of the simulated annealing algorithm and the good global search capability of the genetic algorithm to optimize cluster analysis. The optimal result from sampled data was quickly determined to achieve effective and accurate positioning. These features reduced the non-direct aiming error in the indoor UWB environment. The final experimental results showed that the optimized algorithm significantly reduced noise interference as well as improved positioning accuracy in an NLOS indoor environment with less than 10 cm positioning error.

## Introduction

Strategies for optimization to improve the accuracy of indoor wireless positioning have been widely explored in the past (Yin et al., 2019; Guo et al., 2020; Khalaf-Allah, 2020; Khan et al., 2020; Liu et al., 2020). Positioning accuracy is limited by various obstacles in the indoor environment, especially the NLOS environment which affects signal dispersion and occlusion. Therefore, it is imperative to explore strategies for reducing or eliminating these errors. Currently, the commonly used indoor positioning methods are mainly based on Received Signal Strength Indication (RSSI), Time of Arrival (TOA), Time Difference of Arrival (TDOA) and Angle of Arrival (AOA) (Pahlavan et al., 2002; Yin et al., 2015; Sadowski and Spachos, 2018; Li and Rashidzadeh, 2019). Indoor ultra-wideband (UWB) positioning utilizes a positioning method based on signal arrival time, which estimates the location information of the target point based on trilateral ranging. The positioning accuracy of UWB strategy is higher compared with that of other indoor wireless positioning methods. Notably, UWB can be optimized to further improve its accuracy. Several studies have been conducted on UWB application in indoor positioning. A previous study proposed an incremental smoothing method based on Tukey's kernel function to combine UWB and Pedestrian dead reckoning (PDR) data (Li et al., 2019). The performance of incremental smoothing was then compared with the performance of the optimal fusion algorithm based on EKF (Extended Kalman filter). The finding indicated that real-time positioning was achieved through combination of UWB and PDR, and the two algorithms exhibited high robustness to the intermittent noise of UWB data. A dynamic adaptive covariance Kalman filter based on ultra-wideband positioning and inertial measurement sensor fusion was proposed in a previous study (Briese et al., 2017). The state covariance matrix was adjusted based on absolute acceleration data of the inertial sensor resulting in a good solution for static and dynamic measurement. Furthermore, a low-precision miniature inertial measurement unit (MIMU)/UWB integrated positioning scheme was used for analysis and verification of the integrated system performance, exhibiting a relatively optimal effect (Shi et al., 2017). Moreover, a fusion positioning system based on IMU (Inertial Measurement Unit) and UWB was previously evaluated (Feng et al., 2020). The EKF algorithm based on multiple observation base stations was added to the method to improve the positioning accuracy. In addition, AUM (Approximate uniform motion) and AUAM (Approximate uniform acceleration motion) approximate motion models were used to optimize the positioning results, thereby reducing inaccuracy of positioning data. Analysis was based on the Gaussian noise environment, and further optimization was required in the complex environment. A stable SINS (Strapdown inertial navigation system)/UWB shearer integrated positioning system based on the multi-model intelligent switching method was previously proposed (Yang et al., 2019). The SINS/UWB shearer integrated positioning system was established based on the tightly coupled integration model and the decision tree fault-tolerant model. The findings indicated that the algorithm effectively overcame the positioning errors observed with the tightly coupled model and the decision tree model by exploiting advantages of the two algorithms. A federated EFIR (Equiripple finite impulse response) filter was previous applied in INS/UWB integrated human body positioning (Xu et al., 2018). The federated EFIR filter uses sub-filters to integrate UWB and INS (Inertial navigation system) measurement distance between the reference node and the target person. The optimal navigation solution is determined according to the INS position and the output of the federated EFIR filter. The findings from the study indicated that this method had a higher positioning accuracy compared with that of the traditional federated EKF method. A sensor fusion scheme based on binocular visual odometer (VO)/UWB was previously reported (Zeng et al., 2019). In the study, the adaptive Kalman filtering method was used to fuse the UWB raw distance measurement data and the binocular VO position information. Further, feasibility of the algorithm was verified by collecting the experimental data of two sets of wheeled vehicles. The findings from the study have potential application in solving the limitation of indoor positioning of moving vehicles with inaccurate GPS operation. An integrated positioning method was proposed based on ultra-wideband and improved PDR (Chen et al., 2017). The position data of the two methods were merged to achieve complementary advantages and realize the positioning requirements in complex indoor environments. The findings indicated that this method significantly improves UWB Indoor positioning accuracy. The performance of a complete UWB positioning system based on TWR (Two-way ranging) was previously evaluated by testing various configurations and algorithms to improve the positioning accuracy (Barral et al., 2019). The results showed that use of IEKF (Iterative extended Kalman filter) as the positioning algorithm and K-NN (k-Nearest neighbor) or NN (Neural network) for detection of the NLOS ranging value exhibited the optimal performance in the evaluation of the algorithms. However, the mitigation effect of non-direct aiming effects was not optimal, because they strongly depend on the training algorithm environment, thus they require further improvement. A previous study reported a summary of the reliability of the Gauss-Newton method for ultra-wideband positioning (Wang et al., 2020). Moreover, the effect of the second-order partial derivative on the parameter estimation deviation was evaluated, and a hypothesis test judgment index based on Mahalanobis distance was proposed to explore whether the deviation was significant. The findings indicated that a positioning system with the smallest deviation can be designed by detection of the significant deviation caused by the functional model error. In addition, deep learning method was previously developed for UWB positioning (Poulose and Han, 2020). The method uses long short-term memory (LSTM) network to predict the user's position, and performance of the UWB positioning system was evaluated based on LSTM model. An indoor seamless pedestrian tracking scheme was designed based on least squares support vector machine-assisted unbiased finite impulse response (UFIR) filter (Xu et al., 2019). The position of the inertial navigation system and the ultra-wideband navigation system were integrated based on a loosely coupled combined positioning model. Seamless and reliable indoor pedestrian tracking was achieved through compensation of the position error of the inertial navigation system. A combined positioning method based on ultra-wideband and visual guidance used for location of the AGV by identifying the fixed ArUco code on the AGV was previously designed (Hu et al., 2020). The findings from the study showed that the system was characterized by low technical difficulty, low cost, and higher positioning accuracy for paths without obstacles. Several experiments have been conducted to optimize the UWB transceiver to improve the signal transmission efficiency of UWB. A non-coherent UWB receiver was previously designed by exploiting the cluster-sparsity of the received UWB signal (Sharma et al., 2017). The proposed receiver structure enhanced the signal-to-noise ratio at the receiver output compared with the energy detector (ED) by barring inter-clusters noise accumulation. A simple peak detection based non-coherent UWB receiver was designed in a previous study, which can be used for low data rate WSN and IoT based applications (Sharma et al., 2017a). The proposed receiver divides each data symbol frame duration into smaller multiple time windows. The peak of received signal in each time window is detected independently through threshold comparison. The proposed receiver had better system performance compared with the ED receiver, and it exhibited low power consumption and less system implementation complexity. Joint estimation of TOA and data symbols was previously conducted by exploiting the cluster sparsity of the received UWB signal in the presence of impulsive interference (Sharma et al., 2017b). The proposed receiver structure enhanced the signal-to-noise ratio at the receiver output by minimizing the impulsive interference and barring inter-clusters noise accumulation. An iterative TR (ITR) UWB receiver was designed for joint time of arrival and data symbol estimation in a previous study (Sharma and Bhatia, 2018). The proposed ITR receiver only used single reference pulse for a burst of data symbols. The ITR receiver estimated a new reference pulse from the burst of data symbols' pulses, which further enhanced the signal-to-noise ratio (SNR) of UWB system. The findings indicated that the proposed ITR receiver had high energy efficiency and data rate transmission efficiency with improved SNR relative to that of a TR receiver. UWB positioning is an extensively researched field. However, the positioning accuracy of UWB approach does not meet the optimal accuracy required in industrial application. Therefore, studies should be conducted to explore strategies for improving the software algorithm.

Currently, the positioning accuracy of UWB wireless positioning technology ranges from 0.1 to 1 m. This implies that its accuracy for positioning in a non-line-of-sight environment can be significantly improved. The aim of the present study was to explore a strategy to improve the clustering algorithm for the indoor positioning system based on UWB technology. An improved fuzzy clustering analysis method for indoor wireless positioning optimization was used for large-scale filtering of error and noise, thus rapidly obtaining an optimal positioning value. In the current study, the error form of the TOA trilateral positioning algorithm was evaluated, then the basic positioning accuracy was increased by improving and optimizing the trilateral positioning algorithm. Subsequently, an improved fuzzy c-means (FCM) algorithm was designed to optimize UWB NLOS environment positioning. An annealing evolutionary algorithm (AEA) for optimization was then designed that combines genetic algorithm (GA) and simulated annealing (SA) algorithm to alleviate improper selection of the initial value for the FCM algorithm, which results in easy convergence to a local minimum. The AEA algorithm combines the advantages of GA and SA, thus achieving the global optimal solution within a shorter time, and solves the optimization problem faster and more efficiently. In addition, the results from experimental verification showed that the method designed in the present study exhibited significant optimization effect on UWB indoor positioning. The 0.1 m error exhibited 90% accumulation distribution indicating that the algorithm had high accuracy and positioning stability. Moreover, the error value stabilized within 10 cm. The findings indicate that the method has good theoretical feasibility in positioning optimization field.

## Improved trilateral positioning algorithm to optimize UWB basic positioning

**Figure 1:**
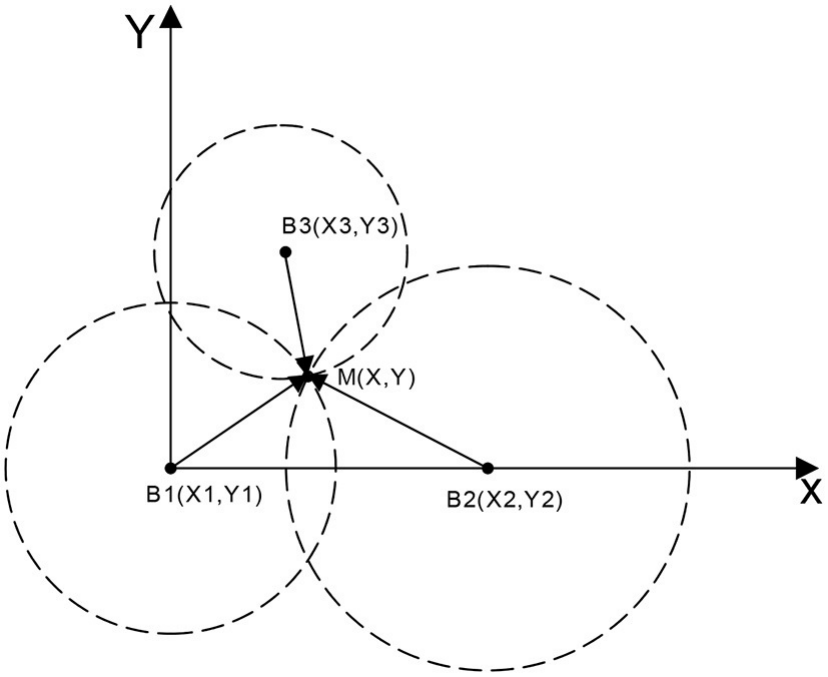
TOA positioning method. **M** represents the positioning tag, **B**_**i**_ indicates the base station node, and the distance between **M** to each base station is denoted *d*_*i*_.

The UWB positioning method used in the study was based on the TOA method, which is a positioning method based on distance. The principle of the method is to obtain the distance between the base station and the tag by determining the time delay from the node to the base station. Subsequently, the specific location of the node is determined and tested using trilateral positioning algorithm (Aditya and Molisch, 2018; Chrabieh, 2020) (Figure 1). TOA positioning uses the high time resolution of the ultra-wideband signal to accurately estimate the distance from the node to the base station by evaluating the signal time delay.

The actual coordinates of the tag **M** can be obtained using the equation below:


(1) $${\text{d}}_{\text{i}}=\sqrt{{\left({\text{x}}_{\text{i}}-\text{x}\right)}^{2}+{\left({\text{y}}_{\text{i}}-\text{y}\right)}^{2}}\mathrm{(i = 1,2,3)}$$

This method exhibits errors in the ranging value during actual positioning due to internal factors and environmental factors. This results in various deviations in positioning. Therefore, the error associated with this method was evaluated in the present study and the algorithm was optimized and improved.

### TOA algorithm error analysis

**Figure 2:**
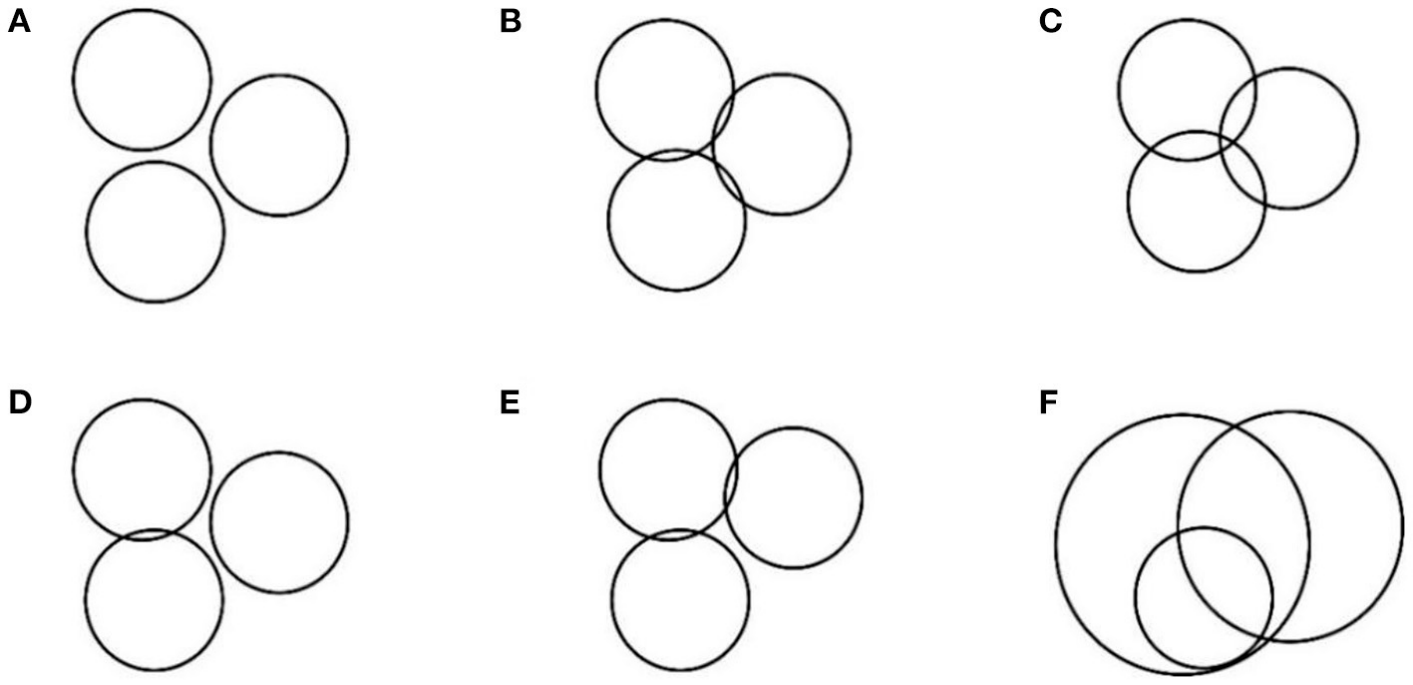
**(A–F)** Possible error forms.

Deviations were observed for the distance measurement between nodes during actual measurement, due to the NLOS error in the indoor positioning environment and the effect of the hardware. Therefore, the circle obtained by trilateral positioning algorithm did not exactly correspond to the representation shown in Figure 1, but had multiple error forms as shown in Figure 2.

**Figure 3:**
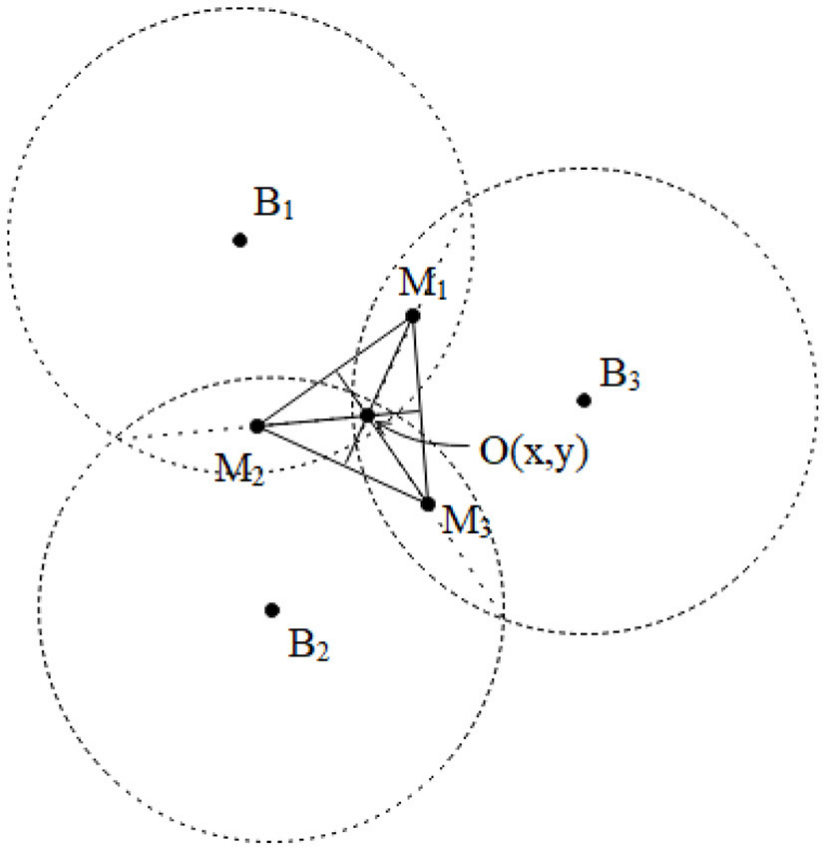
Principle of improved trilateral positioning algorithm.

Analysis indicated that the errors in trilateral positioning algorithm can be attributed to five factors as shown in Figure 2. In the first case, the circles were separated implying that the measured distance was less than the true distance; intersection of two circles as well as three circles implies that the measured value was less than the true distance value, indicating occurrence of a positioning error. Intersection of two circles occurs when the measured distance is less than the true distance resulting in the representation shown in Figure 2B. The representation shown in Figure 2C occurs when the measured distance is greater than the true distance. In this case, the two circles were separated, tangent, or intersected; and the two circles overlapped. The absolute high accuracy shown in Figure 1 does not exist in actual positioning, especially under NLOS environment. Various errors occur during positioning as shown in Figure 3. Therefore, it is imperative to design an algorithm to reduce the error in the trilateral positioning algorithm to minimize the impact on this algorithm. The findings from the experiments in the present study showed that the trilateral positioning algorithm mainly exhibited the form presented in Figures 2B,C in the actual application test. In addition, the results showed that the centroid positioning algorithm can be used to accurately estimate the tag point position by calculating the center point coordinate value of the intersection line between the two circles. Notably, other forms of errors in the figure can be transformed into the form of intersection of two circles using an algorithm, so that the precise tag node position can be estimated through the centroid positioning algorithm.

### Improved trilateral positioning algorithm

The error analysis of the trilateral positioning method indicated that unified algorithm optimization can be performed by conversion of the circles into intersected range circles in pairs for various error manifestations. In this method, the midpoint of the intersection line of the intersecting circles was calculated using the trilateral positioning algorithm, and then the obtained midpoint coordinates were substituted into the centroid positioning algorithm to acquire the position coordinates of the tag node. The principle of the unified optimization algorithm is shown in Figure 3.

In the figure, **M**_**1**_, **M**_**2**_, **M**_**3**_ represent the center points of the intersection lines between the circles, which are also located on the intersection lines of the centers of the circles. The coordinates (*x, y*) of the centroid **O** (the position coordinates of the tag node) were obtained by centroid positioning algorithm using **M**_**1**_, **M**_**2**_, **M**_**3**_ as three known vertices. The error forms of separation and tangents can be converted using an algorithm according to the positioning forms in the presence of errors listed in Figure 2. For example, the radius length of the two separated circles was proportionally increased to achieve intersection, then **M** points were solved in the current study. The algorithm calculation process under separation of the circles is presented in detail in the subsequent section.

**Figure 4:**
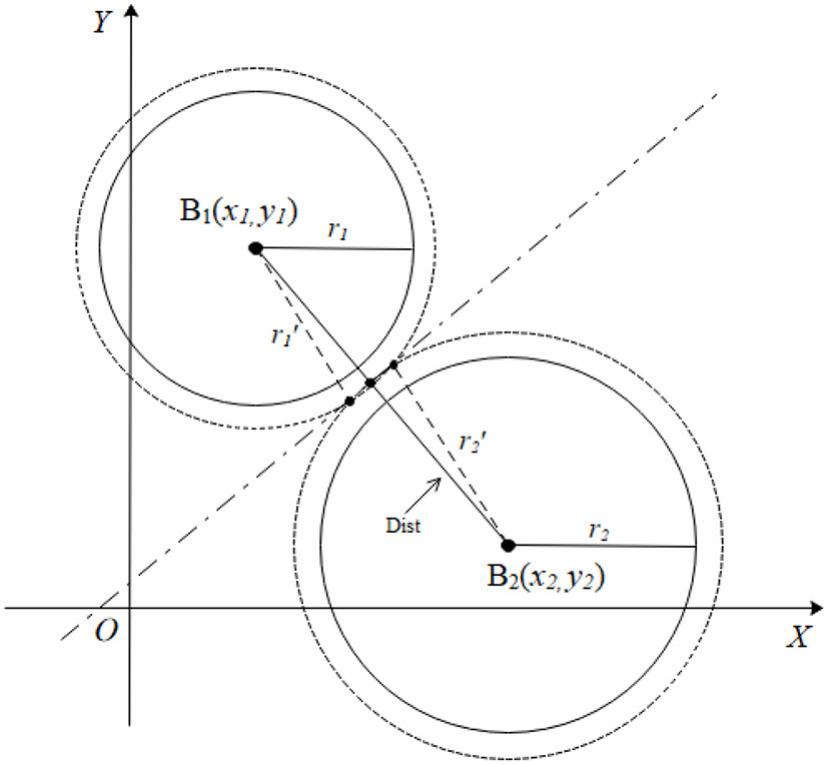
A representation of the separation of two circles.

It was assumed that the actual measured distance values were *r*_1_ and *r*_2_, and the distance between two projection points of the two base station nodes was **Dist** for the projection of the tag node **M** and the two base station nodes **B**_**1**_(*x*_1_, *y*_1_), **B**_**2**_(*x*_2_, *y*_2_) on a two-dimensional plane (Figure 4). The length of **Dist** was calculated using formula (2) below.


(2) $$\text{Dist}=\sqrt{{\left({\text{x}}_{1}-{\text{x}}_{2}\right)}^{2}+{\left({\text{y}}_{1}-{\text{y}}_{2}\right)}^{2}}$$

The circles formed with radius *r*_1_ and *r*_2_ were separated when *r*_1_+*r*_2_<*Dist*, and there was no intersection between them implying that there was an error. The circles *B*_1_ and *B*_2_ were increased in equal proportions until they intersected to effectively eliminate the adverse effects of the error. The proportional coefficient was expressed as shown in formula (3).


(3) $$\text{op}=\;\frac{\text{Dist}}{{\text{r}}_{1}+{\text{r}}_{2}}+0.01$$

In the formula, **op** represents the proportionality coefficient. Notably, 0.01 was added to the expression to ensure that the enlarged circle always intersected, indicating that there was always two intersection points to prevent too small op value that causes separation of the circles. This value can be slightly larger, but if it is too large, the two circles may present an inclusion relationship and will not intersect. Assuming that circles **B**_**1**_ and **B**_**2**_ are enlarged to become circles ${{\text{B}}_{1}}^{\prime }$ and ${{\text{B}}_{2}}^{\prime }$, the corresponding radii will be expressed as ${r_{1}}^{\prime }=r_{1}\times op$, ${r_{2}}^{\prime }=r_{2}\times op$.

**Figure 5:**
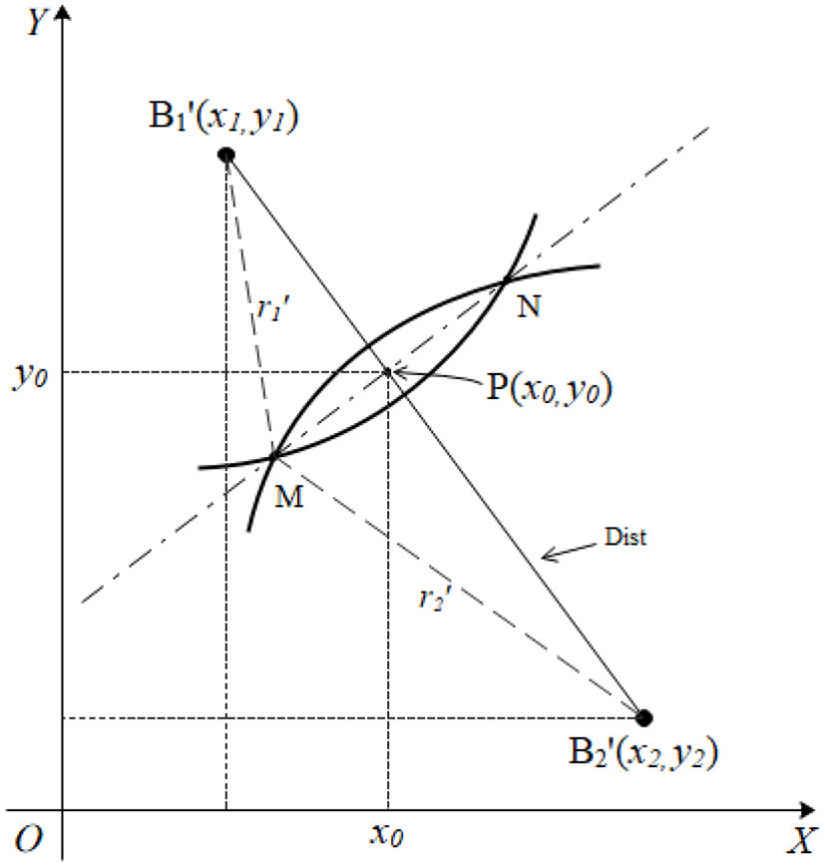
Details of the intersection.

The radii of the two circles are increased according to the proportional coefficient of formula (3) to make them intersect (Figure 4) if the horizontal and vertical coordinates of circles *B*_1_ and *B*_2_ are not equal. The formula indicates that the two intersection points are very close. The intersection of the circles ${{\text{B}}_{1}}^{\prime }$ and ${{\text{B}}_{2}}^{\prime }$ in Figure 4, is enlarged as shown in Figure 5 to facilitate derivation of the formula.

The findings indicate that circle ${{\text{B}}_{1}}^{\prime }$and circle ${{\text{B}}_{2}}^{\prime }$intersect at point **M** and point **N**. The coordinate of the intersection of the line where ${B_{1}}^{\prime }{B_{2}}^{\prime }$ is located and the line where MN is located is presented as *P*(*x*_0_, *y*_0_). The radii of circles ${{\text{B}}_{1}}^{\prime }$ and ${{\text{B}}_{2}}^{\prime }$are ${r_{1}}^{\prime }$ and ${r_{2}}^{\prime }$, respectively, and the distance between the centers of the two circles is **Dist**. The aim is to find the coordinate value of point P(*x*_0_, *y*_0_). The equation set shown below can be obtained from the known variables and the triangle relationship in the figure by setting the length of ${{\text{B}}_{1}}^{\prime }\text{P}$ as *d*_1_, and the length of ${{\text{B}}_{2}}^{\prime }\text{P}$ as *d*_2_:


(4) $$\{\begin{array}{c} {\text{r}}_{1}{{}^{\prime }}^{2}-{\text{d}}_{1}{}^{2}={\text{r}}_{2}{{}^{\prime }}^{2}-{\text{d}}_{2}{}^{2}\\ \text{Dist}={\text{d}}_{1}+{\text{d}}_{2} \end{array}$$

There are two unknown variables in formula (4), namely *d*_1_ and *d*_2_. Formula (5) is obtained after simplifying equation (4):


(5) $${\text{d}}_{1}=\frac{{\text{r}}_{1}{{}^{\prime }}^{2}-{\text{r}}_{2}{{}^{\prime }}^{2}+\text{Dis}{\text{t}}^{2}}{2\text{Dist}}$$

where *d*_1_ represents the length of ${{\text{B}}_{1}}^{\prime }\text{P}$. Similar triangles result in the expression shown below:


(6) $$\frac{{\text{d}}_{1}}{{\text{x}}_{0}-{\text{x}}_{1}}=\frac{\text{Dist}}{{\text{x}}_{2}-{\text{x}}_{1}}$$

Equation (5) can be substituted into equation (6), to obtain the expression presented below.


(7) $${\text{x}}_{0}={\text{x}}_{1}+({\text{x}}_{2}-{\text{x}}_{1})\;\frac{{\text{r}}_{1}{{}^{\prime }}^{2}-{\text{r}}_{2}{{}^{\prime }}^{2}+\text{Dis}{\text{t}}^{2}}{2\text{Dis}{\text{t}}^{2}}$$

The abscissa information of point P can be obtained from the equation, and the line where ${{\text{B}}_{1}}^{\prime }{{\text{B}}_{2}}^{\prime }$ is located has a slope of *k* = (*y*_2_−*y*_1_)/(*x*_2_−*x*_1_). The vertical axis coordinate of point P is then expressed as follows:


(8) $${\text{y}}_{0}={\text{y}}_{1}+\text{k}({\text{x}}_{0}-{\text{x}}_{1})$$

Therefore, the coordinate value of each midpoint is calculated separately. The coordinate information of the tag node can be obtained by substitution equation 8 into equation (9).


(9) $$\left(\text{x},\text{y}\right)=(\frac{\sum_{\text{i}=1}^{\text{k}}{\text{x}}_{\text{i}}}{\text{k}},\frac{\sum_{\text{i}=1}^{\text{k}}{\text{y}}_{\text{i}}}{\text{k}})$$

The step of solving the scale factor **op** can be bypassed when determining the intersection error. A hypothesis statement that compares the size of *r*_1_+*r*_2_ with **Dist** can be added when designing the program. The basic positioning optimization of UWB is completed through the above calculations. Therefore, the FCM algorithm in the cluster analysis algorithm will be introduced in the subsequent section to further optimize the accuracy of UWB indoor positioning.

## Improved FCM algorithm for UWB indoor positioning

### Fuzzy C-means clustering algorithm (FCM)

Fuzzy C-means clustering (FCM) is a commonly used clustering method (Havens et al., 2012; Huang and Chuang, 2012; Lin, 2014; Cardone, 2020). It is a popular form of K-means clustering algorithm based on the fuzzy theory (Im et al., 2020). FCM provides more flexible clustering results compared with the hard clustering of the K-means algorithm. In most cases, the objects in the data set cannot be divided into distinctively separated clusters, so assigning an object to a specific cluster is uncertain, and errors may also occur. Therefore, it is imperative to assign a weight to each object and each cluster, and indicate the degree to which the object belongs to the cluster. Notably, probability-based methods can provide these weights, but sometimes it is challenging to determine a suitable statistical model. Therefore, FCM with natural and non-probabilistic characteristics should be used (Fan and Zhen, 2003).

Assuming **n** data samples are expressed as **X** = {**x**_**1**_, **x**_**2**_, …, **x**_**n**_}, **c**(2 ≤ *c* ≤ *n*) represents the number of data sample types to be divided, {**A**_**1**_, **A**_**2**_, …, **A**_**c**_} represents the corresponding **c** categories and U indicates the similarity classification matrix, the cluster center of each category is expressed as {**v**_**1**_, **v**_**2**_, …, **v**_**c**_}, **μ**_**k**_**(x**_**i**_**)**, which indicates the membership degree of the sample **x**_**i**_ to the class **A**_**k**_ (abbreviated as μ_*ik*_). The objective function **J**_**b**_ can then be expressed as follows:


(10) $${\text{J}}_{\text{b}}\left(\text{U},\text{v}\right)=\sum_{\text{i}=1}^{\text{n}}\sum_{\text{k}=1}^{\text{c}}{\left(\mu_{\text{ik}}\right)}^{\text{b}}{\left({\text{d}}_{\text{ik}}\right)}^{2}$$

where,


(11) $$d_{\text{ik}}=\text{d}\left({\text{x}}_{\text{i}}-{\text{v}}_{\text{k}}\right)=\sqrt{\sum_{\text{j}=1}^{\text{m}}{\left({\text{x}}_{\text{ij}}-{\text{v}}_{\text{kj}}\right)}^{2}}$$

**d**_**ik**_ represents the Euclidean distance, which is used to determine the distance between the i-th sample **x**_**i**_ and the k-th type center point; **m** indicates the sample feature number; **b** represents the weighting parameter, and the value range is 1 ≤ *b* ≤ ∞. FCM clustering method was used to find an optimal classification, so that the classification can result in the smallest function value **J**_**b**_. If the sum of membership values of a sample for each cluster is 1 then formula (12) is obtained:


(12) $$\sum_{j=1}^{c}\mu_{j}(x_{i})=1,\;i=1,2,\ldots ,n$$

Equations (13) and (14) were used to calculate the membership degree **μ**_**ik**_ of the sample **x**_**i**_ to the category **A**_**k**_ and c cluster centers {**v**_**i**_}, respectively:


(13) $$\mu_{\text{ik}}=\frac{1}{\sum_{\text{j}=1}^{\text{c}}{\left(\frac{{\text{d}}_{\text{ik}}}{{\text{d}}_{\text{jk}}}\right)}^{\frac{2}{\text{b}-1}}}$$

Suppose **I**_**k**_ = {*i*|2 ≤ *c* ≤ *n*; *d*_*ik*_ = 0}, for all **i** types, *i*∈*I*_*k*_, μ_*ik*_ = 0 then the following expression is obtained:


(14) $$v_{\text{ij}}=\frac{\sum_{\text{k}=1}^{\text{n}}{\left(\mu_{\text{ik}}\right)}^{\text{b}}{\text{x}}_{\text{kj}}}{\sum_{\text{k}=1}^{\text{n}}{\left(\mu_{\text{ik}}\right)}^{\text{b}}}\;$$

Formulas (13) and (14) can be used to optimize the clustering center, data membership degree and classification. The various cluster centers and the membership degree of each sample to each model class can be theoretically obtained when the algorithm converges, thus completing the fuzzy clustering division. Although FCM has a high search speed, it is a local search algorithm, which is very sensitive to the initial value of the cluster center. The algorithm converges to a local minimum if the initial value is not selected accurately.

### Limitations of traditional FCM clustering algorithm in UWB positioning

FCM algorithm was used to optimize the UWB positioning. A total of 400 sets of data were read per second to obtain the location information of the positioning point based on the high transmission rate and low transmission power of UWB. The obtained data was identified and classified using the FCM algorithm. The obtained cluster center value of each category was expressed as **c**_**i**_(*x*_*i*_, *y*_*i*_), and the coordinates *A*(*x, y*) of the positioning point were obtained using Equation (15). In the equation, *c* represents the number of classification categories based on the clustering algorithm.


(15) $$\text{x}=\frac{\sum_{\text{i}=1}^{\text{c}}{\text{x}}_{\text{i}}}{\text{n}}\;,\;\text{y}=\frac{\sum_{\text{i}=1}^{\text{c}}{\text{y}}_{\text{i}}}{\text{n}}$$

**Figure 6:**
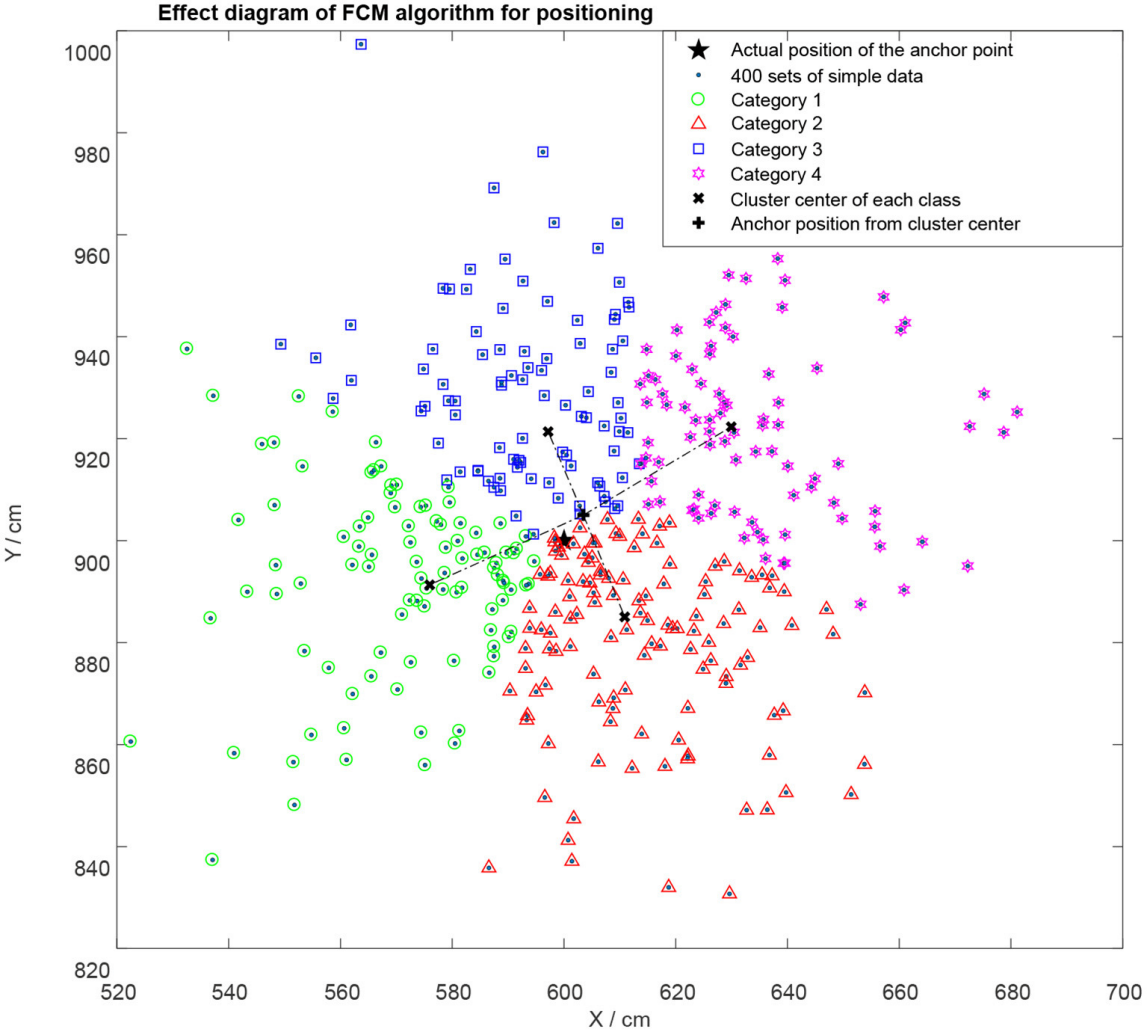
A representation of the four classification effects of FCM algorithm.

The location point (5.00, 3.00) was used as an example to test the positioning performance of the algorithm. The power exponent was set to 2, the maximum number of iterations was set to 10, and the target termination tolerance to 1 × 10^−3^ when category **c** was 4. The positioning effect after the algorithm optimization is shown in Figure 6.

The objective function value **J**_**b**_ = 6.68654 in Figure 6. The results obtained from multiple runs were different, which was attributed to the selected center point of the initial clustering. The cluster center of each category, the calculated position point coordinates and the true coordinates of the point to be located are indicated. The findings showed that a better positioning effect were achieved under the FCM algorithm, but the algorithm optimization also had a certain position error due to its limitations such as easy convergence to local extreme value. The error and algorithm running time were tested under different classification conditions, and the data shown in Table 1 were obtained.

**Table 1:** Optimization efficiency of FCM algorithm under different clustering conditions.

**Cluster point**	**1**	**2**	**3**	**4**	**5**	**6**	**7**	**8**
Average test value	(5.068,2.923)	(5.061,2.929)	(5.045,2.943)	(5.059,2.954)	(5.061,2.939)	(5.056,2.949)	(5.059,2.923)	(5.062,2.935)
Error	0.1027	0.0936	0.0726	0.0748	0.0863	0.0757	0.097	0.0898
Time consumption of algorithm	0.1175	0.1258	0.1182	0.1213	0.1502	0.1346	0.1442	0.1524

The experimental data showed that the UWB positioning under the optimization of the three-category FCM clustering algorithm had relatively high accuracy and small algorithm delay. In the next sections, the algorithm was further modified using the three-category FCM clustering method.

### Improved FMC algorithm based on annealing evolution (AEA-FCM)

FCM algorithm is a local search optimization algorithm, thus it converges to a local minimum if the initial value is not accurately selected. Therefore, the annealing evolution algorithm was used in this study to optimize the FCM clustering algorithm to circumvent this shortcoming. Annealing Evolutionary Algorithm (AEA) integrates simulated annealing algorithm (SA) and genetic algorithm (GA). Therefore, it utilizes SA local search ability and GA global search ability, thus overcoming limitations of poor SA global search ability and low efficiency as well as the poor GA local search ability and prematurity (Krishna, 1999; Huo et al., 2020). The advantages of the two algorithms complement each other, and the outcome is significantly improved through mutation and selection to simultaneously search the solution space. Metropolis criterion was used in the selection hence the advantage of SA algorithm in easily jumping out of a local extremum “trap” was retained. As a result, the algorithm converged to the global minimum more rapidly and efficiently, making it possible to find the global optimal solution faster. The genetic coding method and fitness function were designed according to the specific conditions of the clustering problem, such that the algorithm converged to the global optimal solution effectively and faster.

The algorithm first used genetic algorithm to perform a global search, then the best partial individuals were selected for annealing after a new population was obtained, and ultimately local searches were performed in their respective neighborhoods. Individuals far from the global optimal solution were not searched, which minimized unnecessary searches and markedly reduced the algorithm calculation time.

#### FCM parameter setting

The FCM clustering parameters were set before conducting the calculations. In the present study, the power exponent was set to 2, the maximum number of iterations was set to 30, and the termination tolerance of the objective function to 1 × 10^−5^. All control parameters were then initialized, including setting the population size **sizepop**, annealing temperature cooling coefficient **k**, and initializing **c** cluster centers.

Some parameters of the annealing evolution algorithm were defined using the following code in MATLAB.

**Table:** 

%% Parameters of simulated annealing algorithm	
q =0.2;	% Cooling coefficient
T0=100;	% Initial temperature
Tend=1;	% Final temperature
%% Define the genetic algorithm parameters	
sizepop=10;	% Number of individuals
MAXGEN=7;	% Maximum number of generations
NVAR=m*cn;	% Dimension of the variable
PRECI=5;	% Precision of variables
GGAP=0.95;	% Generation gap
pc=0.7;	% Crossover probability
pm=0.01;	% mutation probability

#### Individual coding

The parameters to be optimized in the genetic clustering algorithm were **c** initial cluster centers. Binary coding was used for the optimization process. Each chromosome utilized **c** cluster centers to create the initial population **Chrom**. The number of variables to be optimized for an m-dimensional sample vector was *c* × *m*. Assuming that each variable used a k-bit binary code, then the chromosome was a binary code with a length *c* × *m* × *k*. The area descriptor **FieldD** was then established to convert the binary matrix **Chrom** into real values. In the present study, the following expression was used:


(16) $$\text{FieldD}={\left[\begin{array}{cccc} \text{PRECI} & \text{PRECI} & \cdots & \text{PRECI}\\ \text{lb} & \text{lb} & \cdots & \text{lb}\\ \text{ub} & \text{ub} & \cdots & \text{ub}\\ 1 & 1 & \cdots & 1\\ 0 & 0 & \cdots & 0\\ 1 & 1 & \cdots & 1\\ 1 & 1 & \cdots & 1 \end{array}\right]}_{7\times \text{NVAR}}$$

where the **FieldD** dimensionality **NVAR** was determined by the dimensionality of the input value and the number of clustering categories, **PRECI** represents the defined binary digits of the variable, and **lb** and **ub** represent the lower and upper bounds of the input value, respectively.

#### Fitness function design

Each individual utilized **J**_**b**_ obtained by formula (10) as the objective function. A small **J**_**b**_ indicated a high fitness value of the individual. The fitness distribution function *f*_*i*_ = *ranking*(*J*_*b*_) in the MATLAB genetic algorithm toolbox was used for the fitness function design. The individual target values **J**_**b**_ were sorted in ascending order, and the column vector containing the corresponding individual fitness value **f**_**i**_ was determined. The fitness value was calculated using Equation (16) shown below: where, **sp** represents the selected pressure difference used to determine the offset or selection intensity. The fitness value of each individual was calculated according to its position **Pos** in the sorted population.


(17) $$\text{FitnV}\left(\text{Pos}\right)=2-\text{sp}+\frac{2\left(\text{sp}-1\right)\left(\text{Pos}-1\right)}{\text{sp}-1},\;\text{sp}\in [1,2]$$

#### Genetic operator

##### Selection operator

Random traversal sampling was used to select operators, and the probability of each selected individual was determined using the previously determined fitness value **FitnV**. Individuals with high fitness after conducting repeated experiments had higher chance of being inherited into the next generation population when the generation gap between individuals (the ratio of individuals selected in the next generation) was set to 0.95. The individuals with low fitness had low probability of being inherited into the next generation population. The aim of selection operation was to select some individuals from the parent population to be inherited in the next generation population. The selection process adopted the roulette selection method. The probability that individual **x**_**i**_ in the population was selected if the population size was **n** was determined by the formula shown below:


(18) $$\text{p}\left({\text{x}}_{\text{i}}\right)=\frac{{\text{f}}_{\text{i}}({\text{x}}_{\text{i}})}{\sum_{\text{j}}^{\text{n}}{\text{f}}_{\text{i}}({\text{x}}_{\text{j}})}\;$$

This method ensures that a high fitness corresponds to a high probability of individuals being selected.

##### Crossover and mutation operator

**Figure 7:**
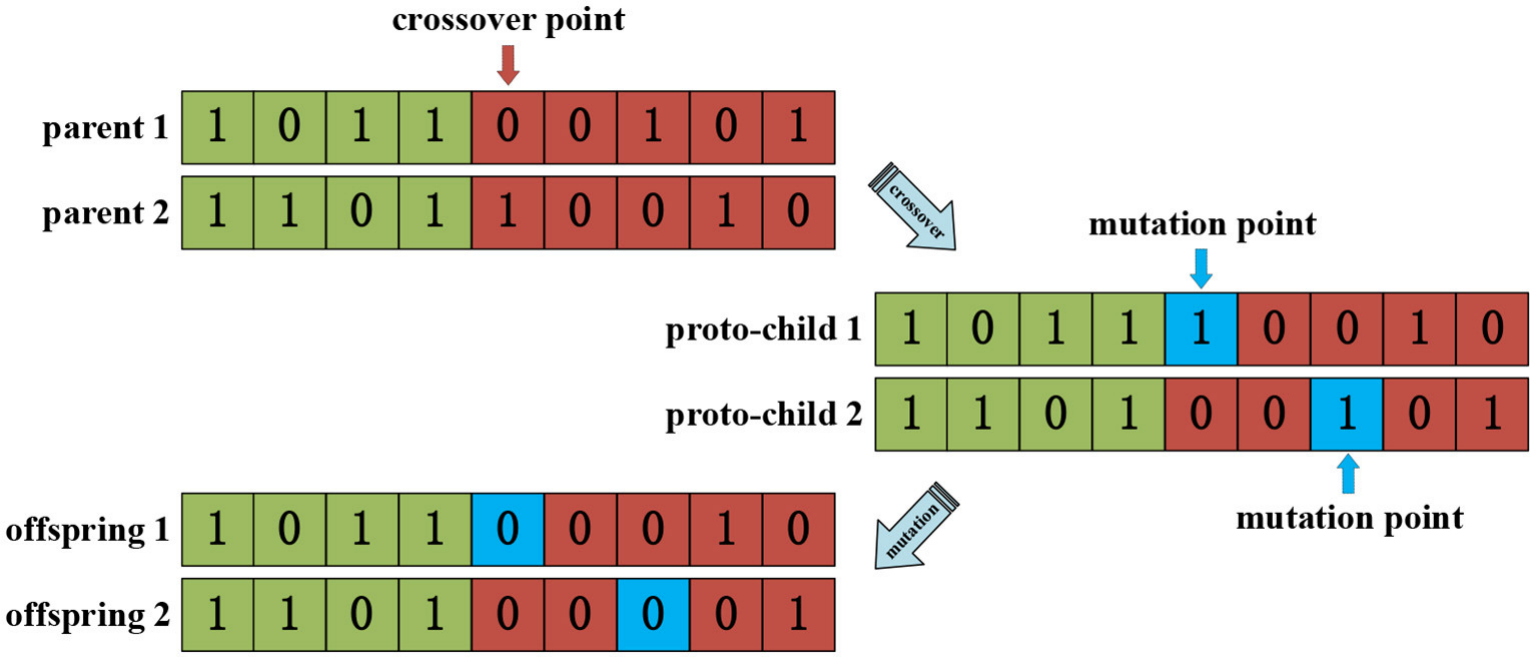
Crossover and mutation processes using the genetic algorithm.

A single-point crossover method was used in the crossover operator to perform genetic recombination on the individuals selected by the selection operator according to probability. The mutation operator adopted the basic bit mutation operator. The number of mutated genes was generated with a certain probability using the mutation operator, and the mutated genes were selected using random methods. For the individual represented by the binary coded symbol string, if the original gene on a certain locus that should be mutated was 0, it was changed to 1. On the contrary, if the original gene value was 1, it was changed to 0. The operating principle of binary crossover and mutation is shown in Figure 7.

#### Combination of genetic algorithm and simulated annealing algorithm

Traditional genetic algorithms have a limitation of premature convergence. This implies that a few individuals in the evolutionary group had superior fitness function value compared with other individuals, leading to premature convergence of the evolutionary process. Therefore, annealing of excellent individuals was utilized to induce excellent individuals to produce some similar new solutions in respective neighborhoods. This minimizes population occupation by very few excellent individuals and prevented the algorithm from premature convergence. The annealing operator was used in the present study to further optimize the algorithm, with the process presented below:

The first nAnneal individuals for annealing were selected after sorting the new population generated by the genetic algorithm as shown in the expression below:


(19) $$\text{nAnneal}\;=\;\text{round}(\frac{\text{sizepop}}{2\text{R}})$$

where, round indicates approximating the outcome using rounding method, and R represents the upper limit of the number of new solutions accepted in the inner loop of the algorithm.

Each individual was then annealed individually. The total number of new solutions obtained by annealing is shown below:


(20) $$\text{nPopAnn}=\sum_{\text{i}=1}^{\text{nAnneal}}\text{r}(\text{i})\;$$

where, **r(i)** indicates the number of new solutions accepted by the i-th individual within **L** iterations. *r*(*i*) ≤ *R*, so *nPopAnn* ≤ *sizepop*/2. *popSize*/2 was used to prevent the annealing-generated individuals from occupying the entire population and from entering a local minimum.

The new solutions were then added to the original population, and the first **sizepop** after sorting was selected for the next step of calculation.

**Figure 8:**
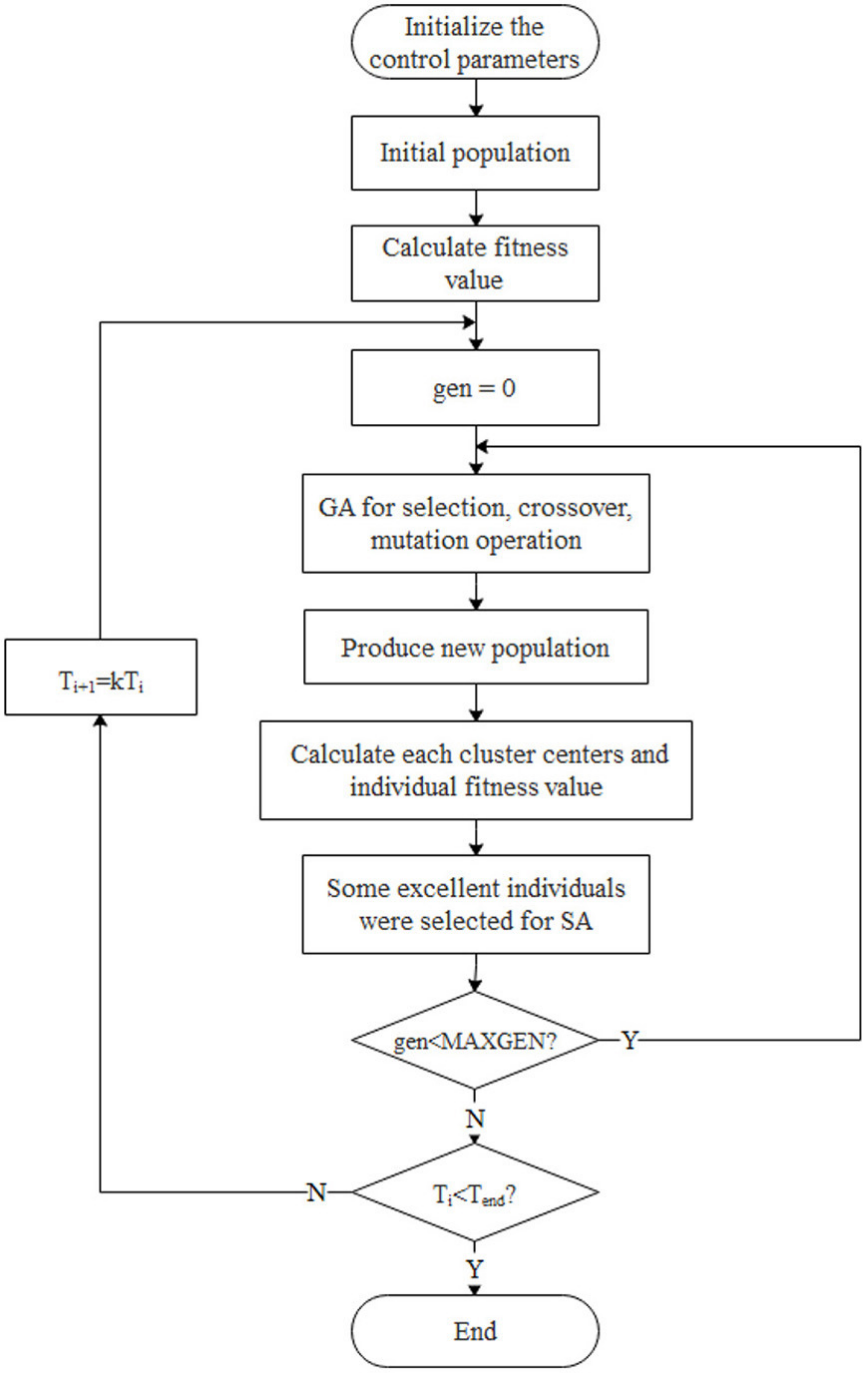
A schematic representation of the improved AEA algorithm.

The improved AEA algorithm workflow is shown in Figure 8.

## Nomenclature

### Experimental results and analysis

**Figure 9:**
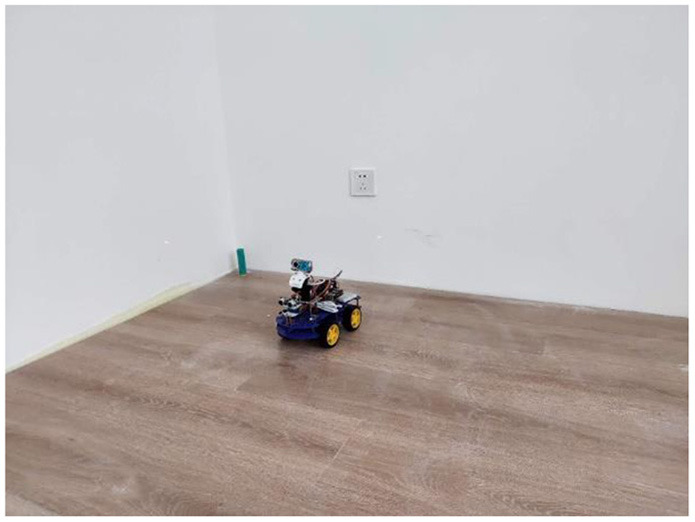
The experimental site.

**Figure 10:**
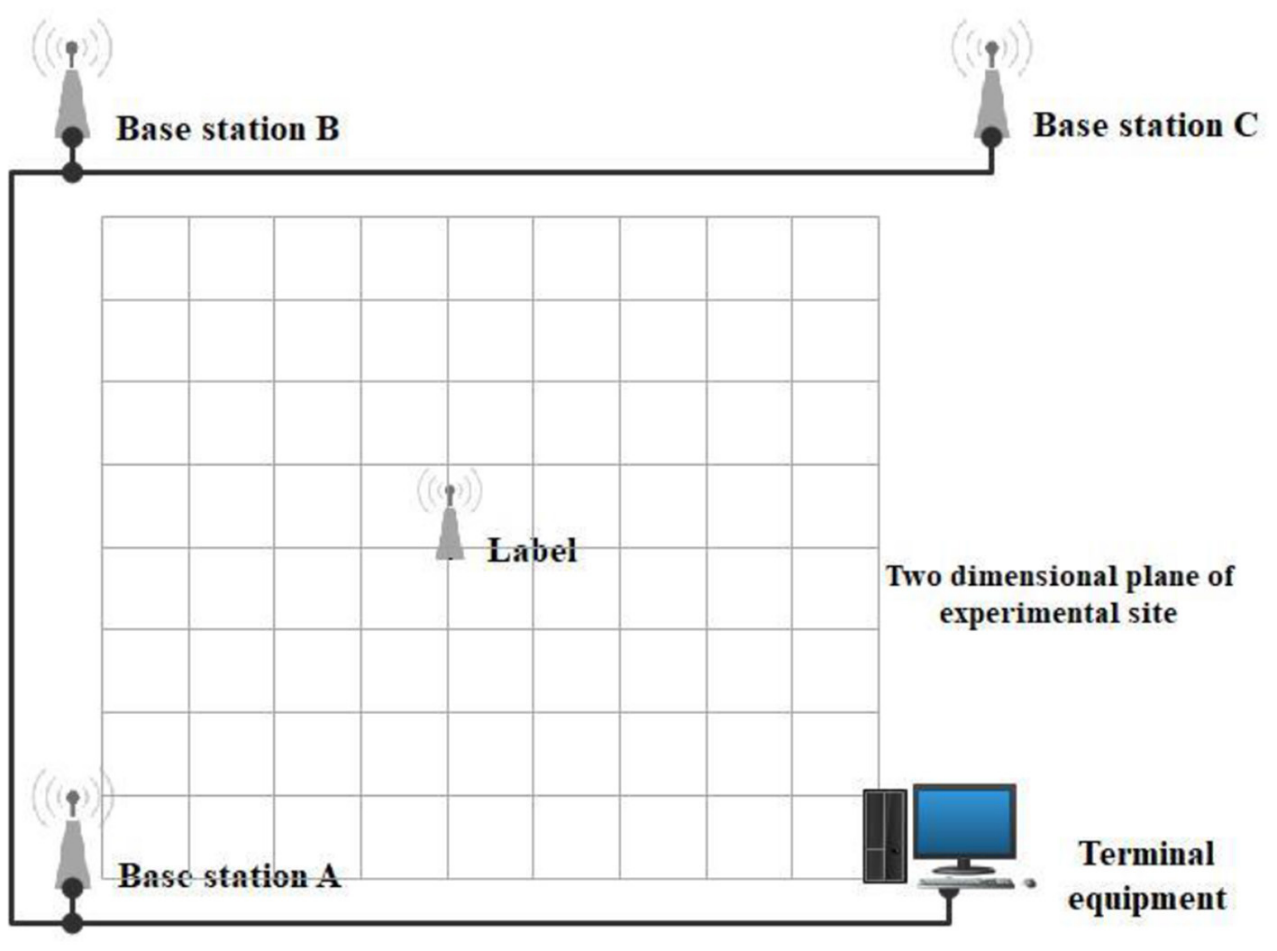
A representation of the simulation process at the experimental site.

**Figure 11:**
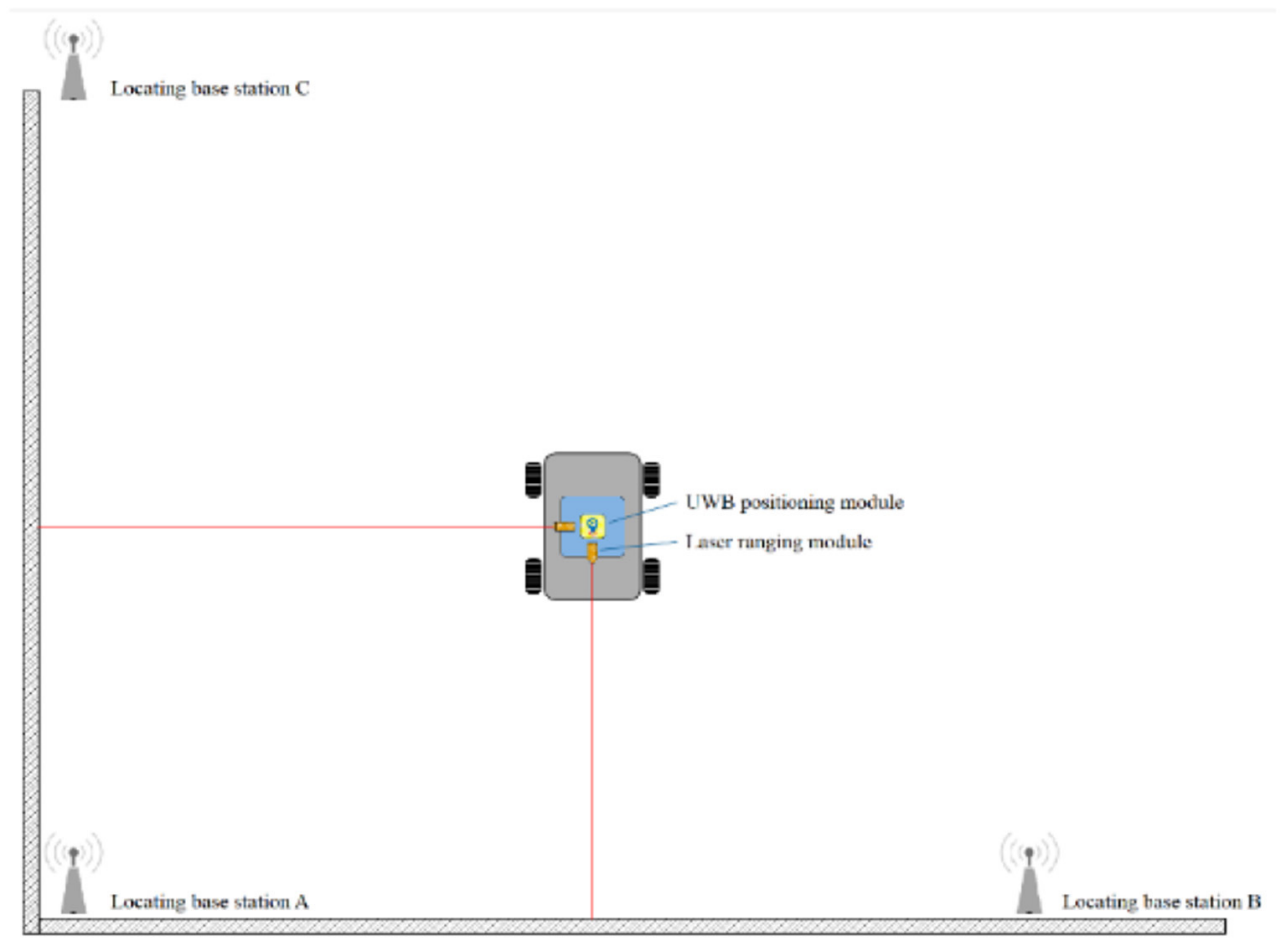
A representation of the experimental simulation.

The experimental site is presented in Figure 9. The experimental site was a flat ground. The *xoy* plane represented the positioning node located in the horizontal plane, which was used to establish a two-dimensional rectangular coordinate system in order to simplify the calculation during the experimental process. The *x* and *y* coordinates of the positioning node were analyzed during the experiment (Figure 10). Laser ranging modules were installed on the tag platform, and two laser ranging modules were used to obtain the real-time coordinate information of the tag. The positioning tag and the ranging module were connected to the mobile station by a rotating shaft to ensure that the two laser ranging modules were always perpendicular to the wall (Figure 11).

**Figure 12:**
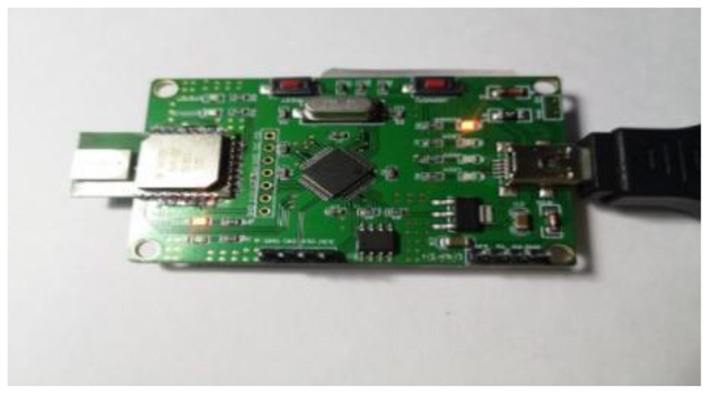
UWB module used in the present study.

DW1000 chip produced by *DecaWave* was used as positioning module in the experiment. It contained 3 base stations and 1 positioning tag. The positioning module supported data rates of 110 kbps, 850 kbps and 6.8 Mbps with an effective transmission distance of 300 meters. The actual module is presented in Figure 12.

**Figure 13:**
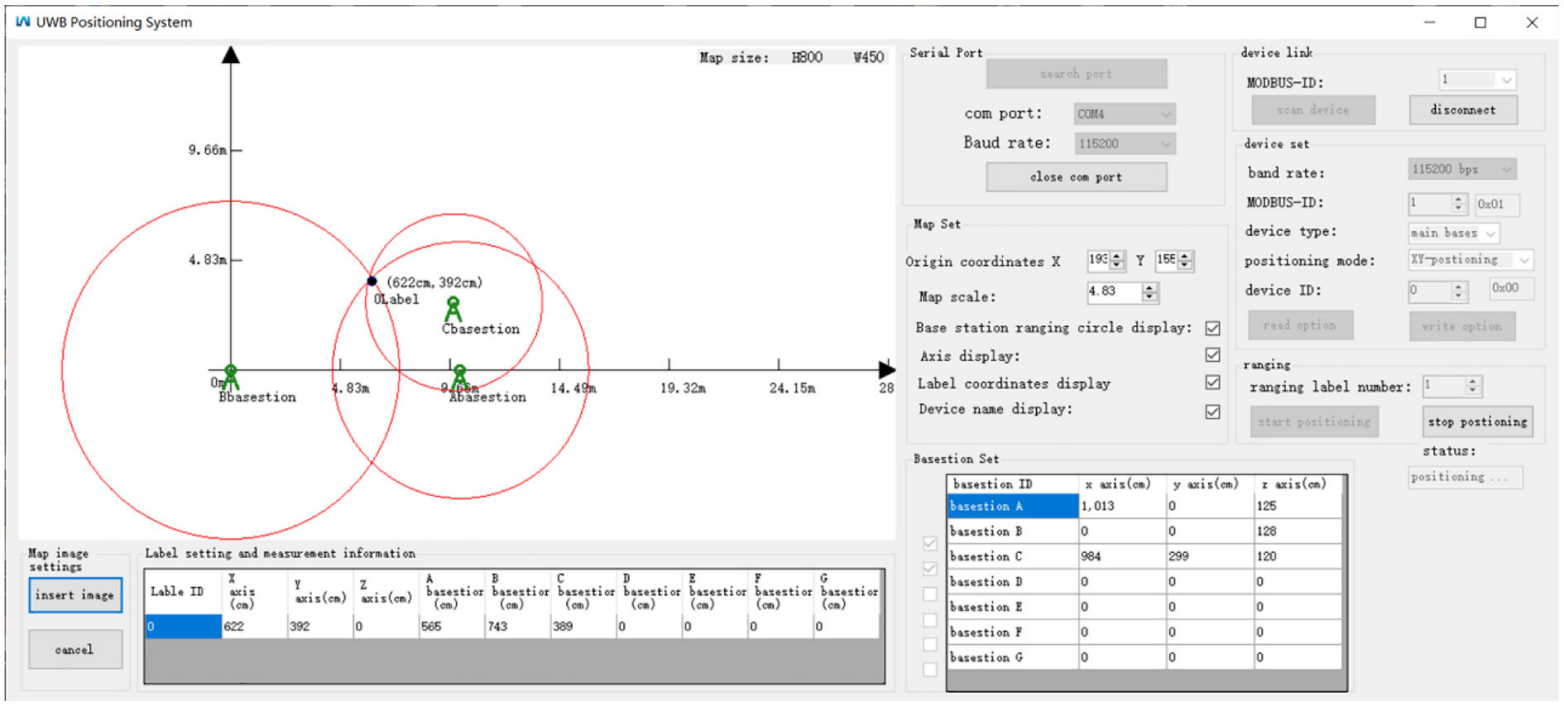
A schematic representation of the upper computer interface.

The computer tools used in the experiment were compiled in *C#* programming language. The main base station connected to the computer was used for transmission and reception of the positioning data. In addition, the base station displayed the relative position coordinates of the tag and the distance to each base station in real time. The interface is shown in Figure 13. The interface displayed the ranging circles of each base station according to the trilateral measurement algorithm under static positioning.

**Figure 14:**
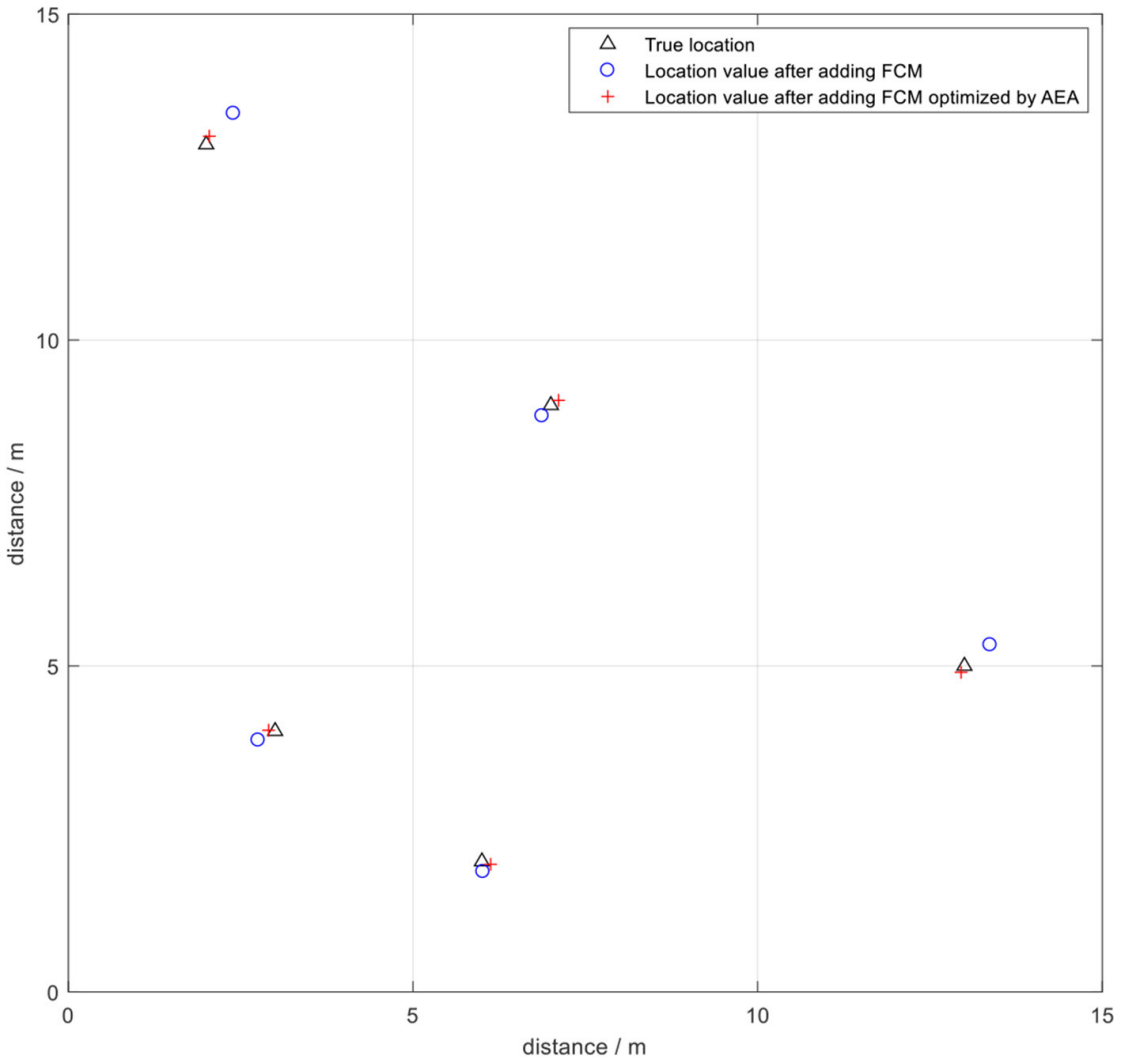
Schematic diagram showing the static positioning.

A number of fixed points in the experimental site were selected to place positioning tags to determine the positioning accuracy of the UWB indoor wireless network positioning system and the optimization performance of the clustering algorithm in a static environment (as shown in Figure 14). Subsequently, each algorithm was tested separately, then the measured data were analyzed and compared.

**Figure 15:**
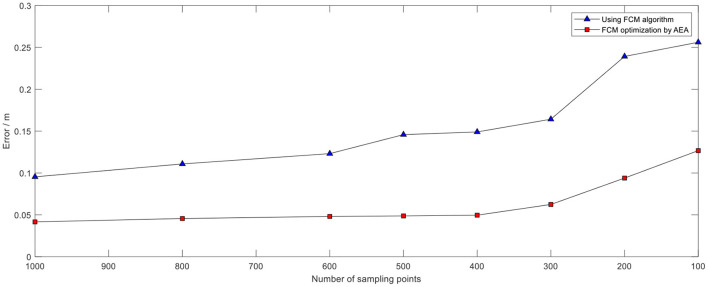
Comparison of errors under the two algorithms under different sampling points.

The positioning data exhibited an error of ±1 m in UWB positioning without the optimization algorithm due to effects of the hardware and indoor environment. The error was presented as a Gaussian distribution around the actual value. Determination of the number of samples points is an important step if clustering algorithm is used to optimize wireless indoor positioning. The appropriate sampling points should be selected to minimize the time the algorithm takes while maintaining high accuracy. A summary of some test data is presented in Table 2. The results indicated that a higher number of sampling points was correlated with a smaller positioning error value. However, the time taken by the algorithm gradually increased with increase in number of sampling points. Analysis of the data showed that the positioning error was smaller under AEA-FCM algorithm optimization, and the overall accuracy was increased by about 35.17% compared with the value before optimization. The positioning error using the FCM algorithm without optimization for 400 sampling points was about 16.23 cm, and the algorithm took approximately 130 ms to converge. The positioning error decreased to 9.96 cm when the annealing evolution was used to optimize the FCM algorithm, and the algorithm took about 280 ms to converge. The accuracy of the optimized algorithm was increased by 38.63% compared with that of the algorithm before optimization. In addition, the algorithm consumed relatively less time with 400 sampling points compared with the time taken by the algorithm before optimization. This implies that the algorithm had optimal efficiency and relatively better positioning effect with 400 sampling points (Figure 15).

**Table 2:** Comparison of algorithm performance under different number of sampling points.

**Number of sampling points**		**10,00**	**800**	**600**	**500**	**400**	**300**	**200**	**100**
FCM algorithm without optimization	Calculated value	(7.945, 10.888)	(7.942, 10.883)	(8.066, 10.873)	(7.932, 10.871)	(7.922, 11.127)	(8.121, 10.889)	(7.846, 10.817)	(8.166, 11.195)
	error	0.1354	0.1408	0.1491	0.1558	0.1623	0.1742	0.1792	0.1931
	Consumption time	0.1426	0.1392	0.1388	0.1371	0.1354	0.1349	0.1307	0.1336
After optimization by annealing evolution algorithm	Calculated value	(8.034, 11.024)	(8.049, 11.022)	(8.040, 11.021)	(8.038, 11.023)	(8.042, 11.050)	(8.051, 11.025)	(8.063, 11.056)	(8.069, 11.066)
	error	0.0852	0.0873	0.0922	0.0967	0.0996	0.1162	0.1257	0.1382
	Consumption time	0.5869	0.4732	0.3898	0.3246	0.2828	0.2732	0.2475	0.2414
Percentage increase in accuracy	37.08%	38.00%	38.16%	37.93%	38.63%	33.30%	29.85%	28.43%

**Figure 16:**
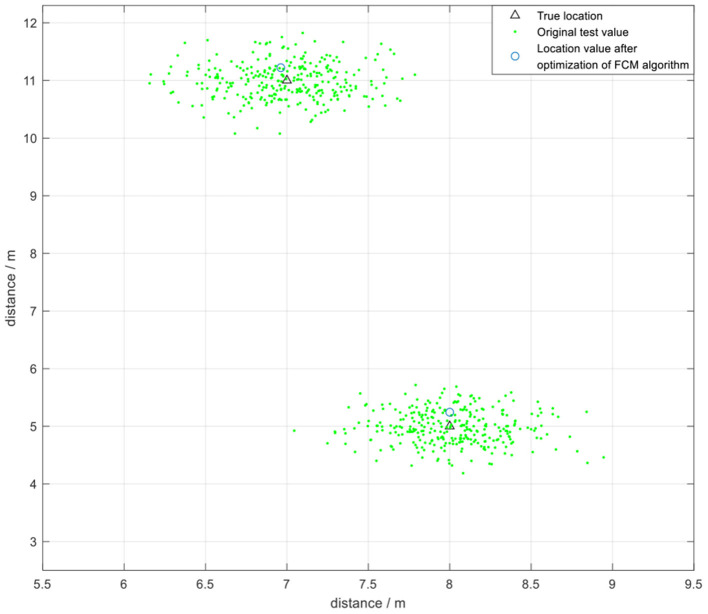
The positioning effect under FCM algorithm without optimization.

The positioning data was collected once every 500 μs during the experiment, and 400 samples were collected once every 200 ms for analysis owing to the high transmission rate of UWB technology. The FCM algorithm was initially used to cluster the data and obtain the optimized clustering center. Two coordinate points (7,11) and (8,5) were selected for testing (Figure 16). The test error was approximately 20 cm. The findings showed that FCM algorithm, which is a local search optimization algorithm, is highly effective for application in indoor positioning. However, the algorithm can easily converge to a local minimum if the initial value is not accurately selected, resulting in increase in positioning instability.

**Figure 17:**
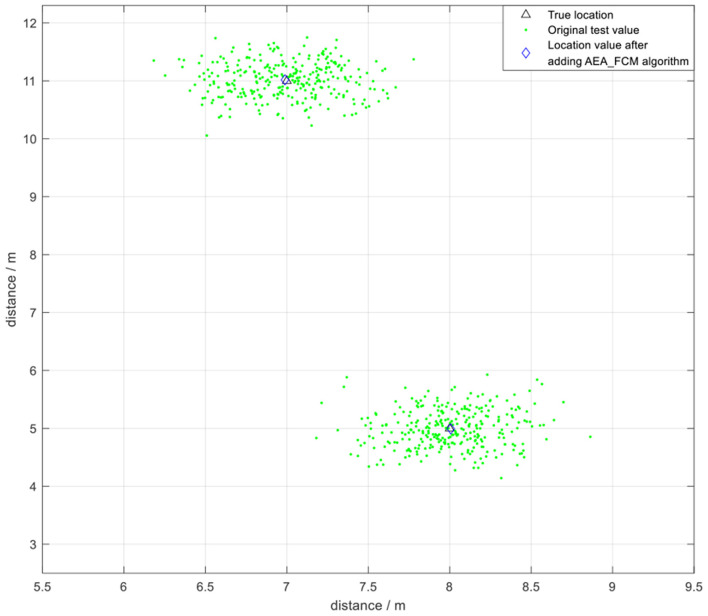
The positioning effect under AEA_FCM algorithm optimization.

**Figure 18:**
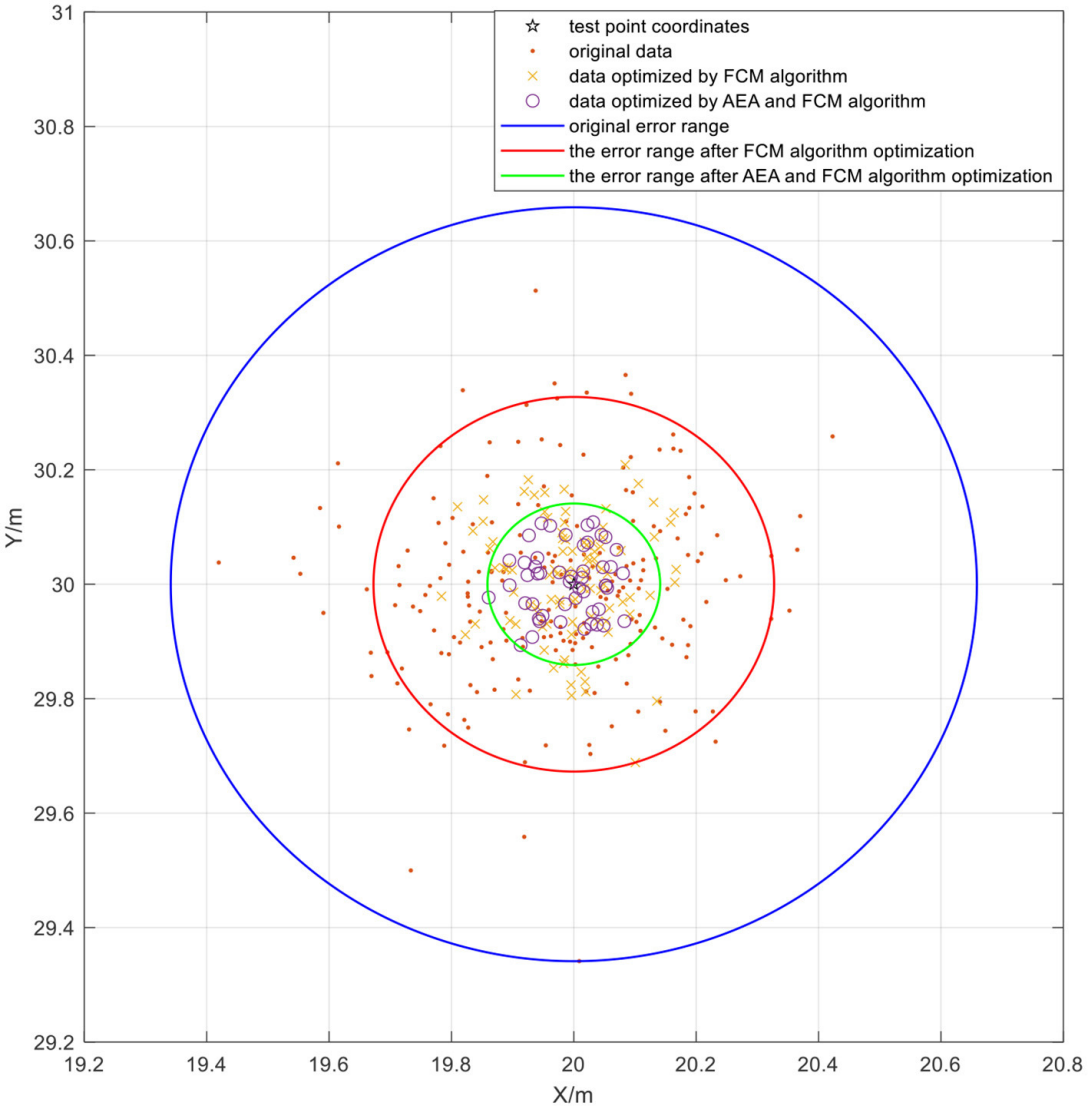
Comparison of positioning effects under application of the two algorithms.

Subsequently, annealing evolution algorithm was used to optimize FCM clustering. The AEA-FCM algorithm exhibited good optimization effect on wireless positioning after repeated tests. The accuracy after optimization increased by 37.12% compared with the positioning algorithm using FCM without optimization, and the accuracy increased by 52.73% compared with the accuracy of the original UWB positioning. The positioning error was stabilized within 10 cm. The positioning effect is shown in Figure 17. Multiple positioning points were selected for testing, and the mean square error of the test results was compared. The results showed that the positioning mean square error optimized by the algorithm was controlled below 3 × 10^−3^, indicating a significant optimization effect (Table 3). Point (7,11) was sampled to demonstrate the effect of optimization. The positioning error range of UWB positioning when using FCM algorithm alone was compared with that of the FCM algorithm optimized by AEA as shown in Figure 18. The powerful local search ability of simulated annealing algorithm and the powerful global search ability of genetic algorithm effectively solved the cluster center value in real time. The findings showed that combination of the two algorithms improved the positioning stability as well as markedly increased the positioning accuracy.

**Table 3:** Mean square error under the two algorithms.

**Anchor coordinates**	**FCM algorithm**		**FCM algorithm optimized by AEA**	
	**locator data**	**MSE**	**locator data**	**MSE**
(2.00, 3.00)	(1.901, 3.093)	0.0092	(1.964, 3.047)	0.0018
(6.00, 5.00)	(6.105, 5.102)	0.0107	(6.052, 5.043)	0.0023
(8.00, 9.00)	(8.096, 8.889)	0.0108	(8.047, 8.957)	0.0020
(14.00, 11.00)	(14.123, 10.911)	0.0115	(13.954, 10.955)	0.0021
(15.00,16.00)	(15.094, 16.118)	0.0114	(15.057, 16.042)	0.0025
(20.00, 19.00)	(20.127, 19.081)	0.0113	(19.943, 19.044)	0.0026
(23.00, 22.00)	(23.131, 22.129)	0.0169	(23.058, 21.967)	0.0029

**Figure 19:**
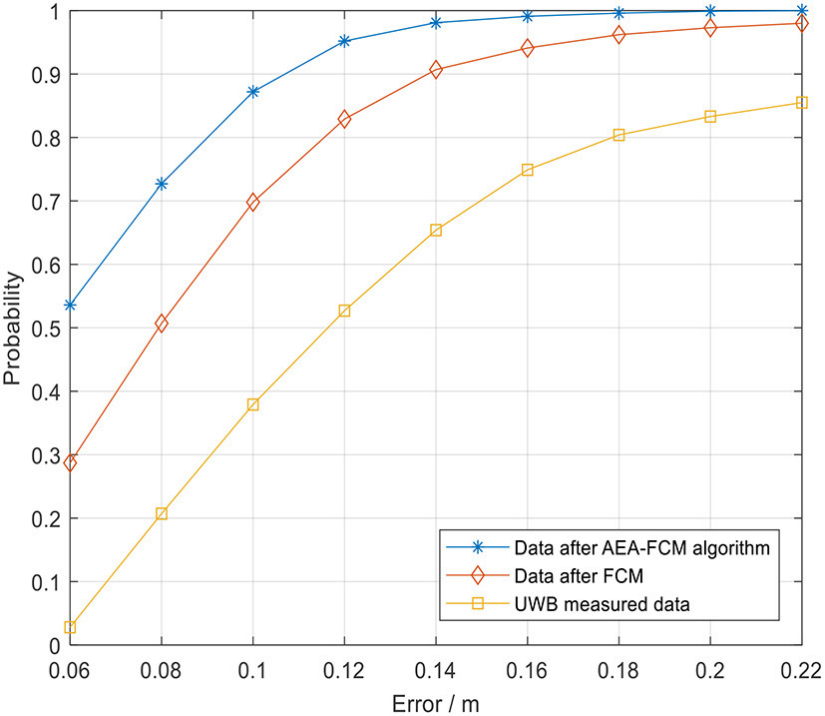
Cumulative distribution of positioning error.

The cumulative distribution function (CDF) of the positioning error is presented in Figure 19. The abscissa represents the positioning error, and the ordinate represents the probability that the error is smaller than or equal to the abscissa value. The results indicated that the 0.1 m error cumulative distribution of UWB positioning without algorithm optimization was about 40% under indoor environment. The error after optimization of the FCM algorithm exhibited a local minimum point due to poor selection of the initial value, resulting in about 70% of 0.1 m error cumulative distribution. The blue line in the figure represents the results obtained after optimization using the fusion algorithm proposed in the current study. The 0.1 m error cumulative distribution was approximately 90%, indicating high accuracy and positioning stability of the algorithm. Notably, the error was highly stable within 10 cm. In summary, the findings indicate that UWB positioning using FCM algorithm has some shortcomings. However, combination of FCM algorithm with AEA algorithm significantly improved the positioning accuracy and markedly increased the positioning stability.

## Conclusion

In the present study, the effect of NLOS error on positioning accuracy was evaluated to improve the accuracy of indoor positioning system based on UWB. An optimization algorithm established by combining AEA and FCM algorithms was proposed and an UWB positioning test system was established. The experimental results showed that the algorithm had the optimal positioning effect when positioning data from 400 sampling points were collected every 200 ms. In addition, the error was significantly reduced by 37.12% compared with that obtained using the FCM algorithm without optimization. The positioning results for the combined algorithm showed better robustness and higher accuracy. The 0.1 m error cumulative distribution of the optimized algorithm was approximately 90%, and the error value was stable within 10 cm. This implies that this method has good theoretical feasibility. The positioning algorithm in the current study can be used in indoor object positioning, elderly condition monitoring as well as prevention of object theft. Further experiments should be conducted in larger and more complex environments to further evaluate the performance of the algorithm. Multi-label parallel location should be explored to improve the response speed and reduce the delay. Further, FPGA hardware acceleration can be used to improve the operation speed of the algorithm.

## Data Availability Statement

The raw data supporting the conclusions of this article will be made available by the authors, without undue reservation.

## Author contributions

HG: conceptualization, methodology, writing–reviewing, and editing. ML: data curation and writing–original draft preparation. XZ: visualization and investigation. XG: software and validation. QL: investigation resources. All authors contributed to the article and approved the submitted version.

## Conflict of interest

The authors declare that the research was conducted in the absence of any commercial or financial relationships that could be construed as a potential conflict of interest.

## Publisher's note

All claims expressed in this article are solely those of the authors and do not necessarily represent those of their affiliated organizations, or those of the publisher, the editors and the reviewers. Any product that may be evaluated in this article, or claim that may be made by its manufacturer, is not guaranteed or endorsed by the publisher.
