# Supplementary figures and images for: UWB indoor positioning optimization algorithm based on genetic annealing and clustering analysis
Source: Front Neurorobot. 2022 Jul 26;16:715440. doi: 10.3389/fnbot.2022.715440 (PMC9360920; doi:10.3389/fnbot.2022.715440)

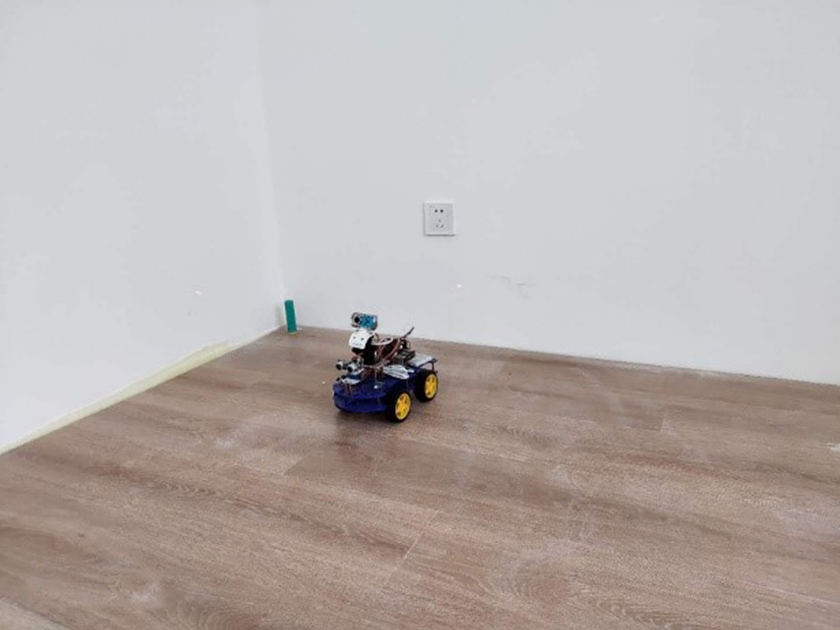

Supplement: Supplementary file 1 [file Image_1.jpg]

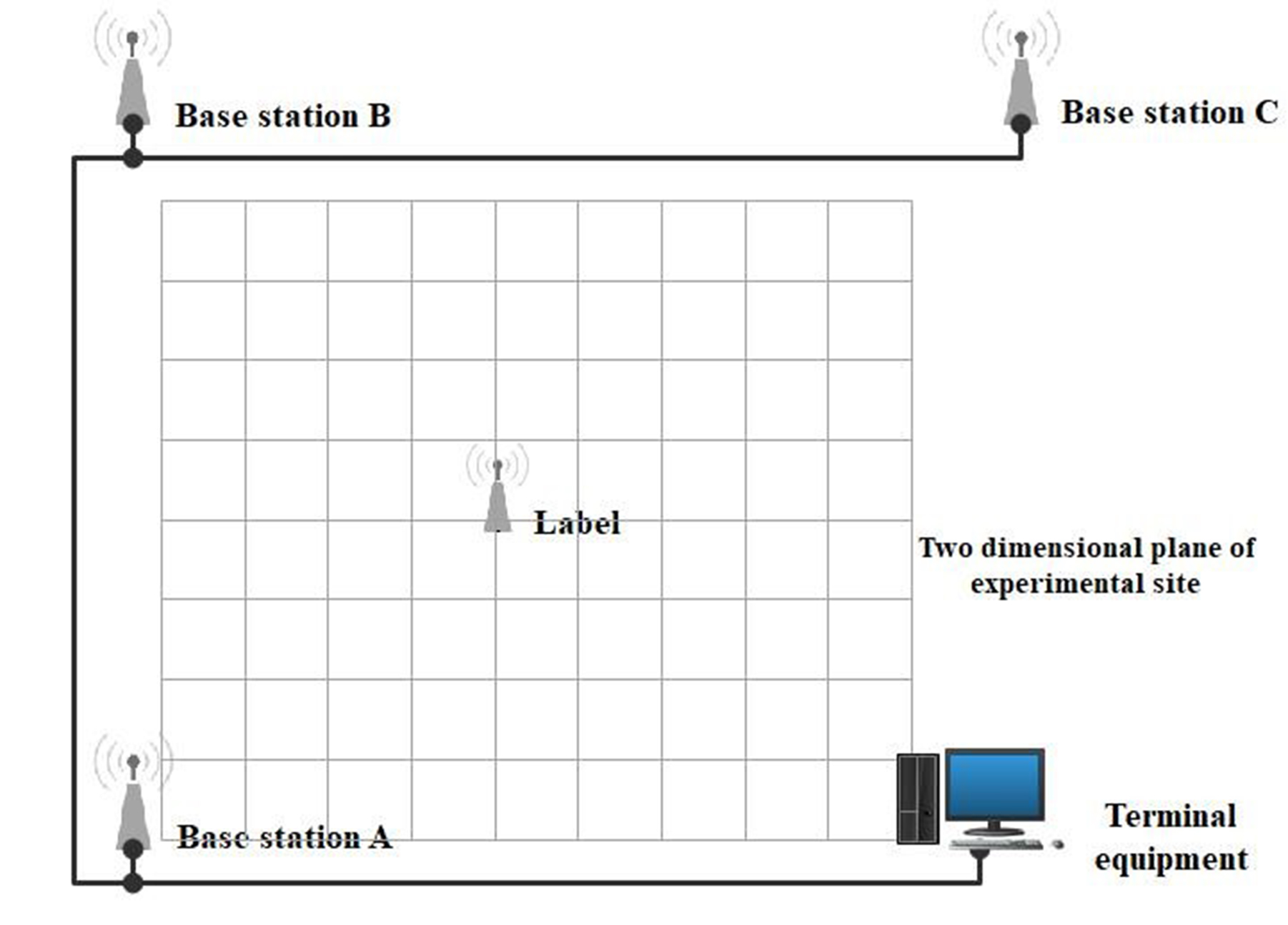

Supplement: Supplementary file 2 [file Image_2.jpg]

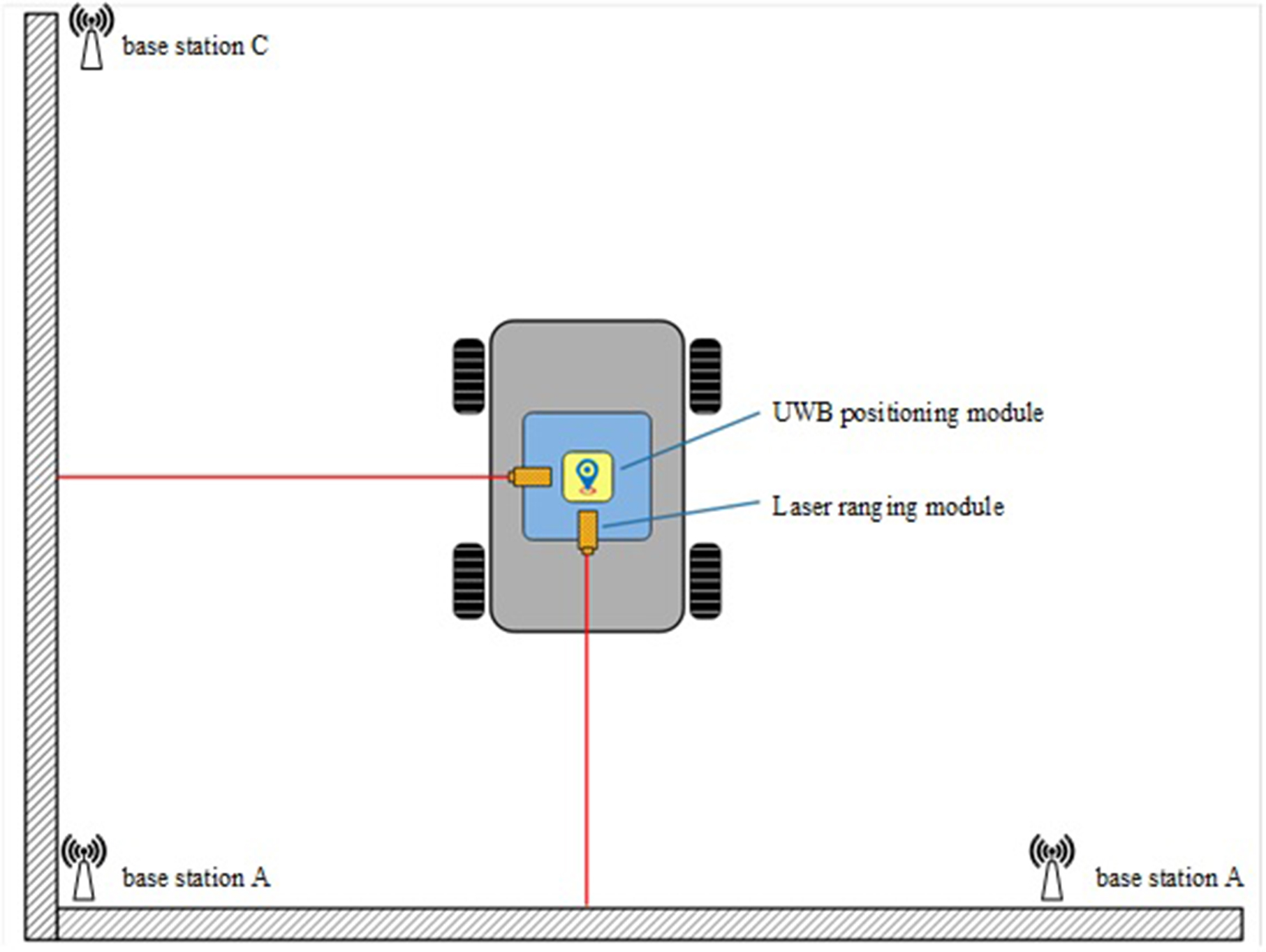

Supplement: Supplementary file 3 [file Image_3.jpg]

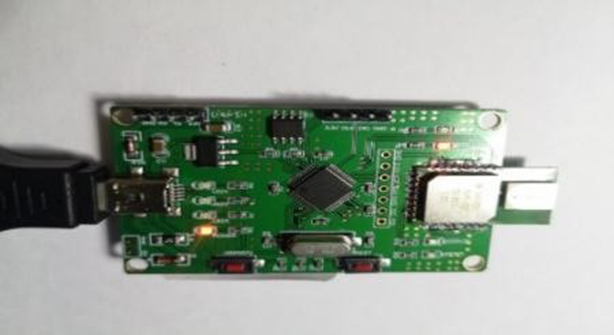

Supplement: Supplementary file 4 [file Image_4.jpg]

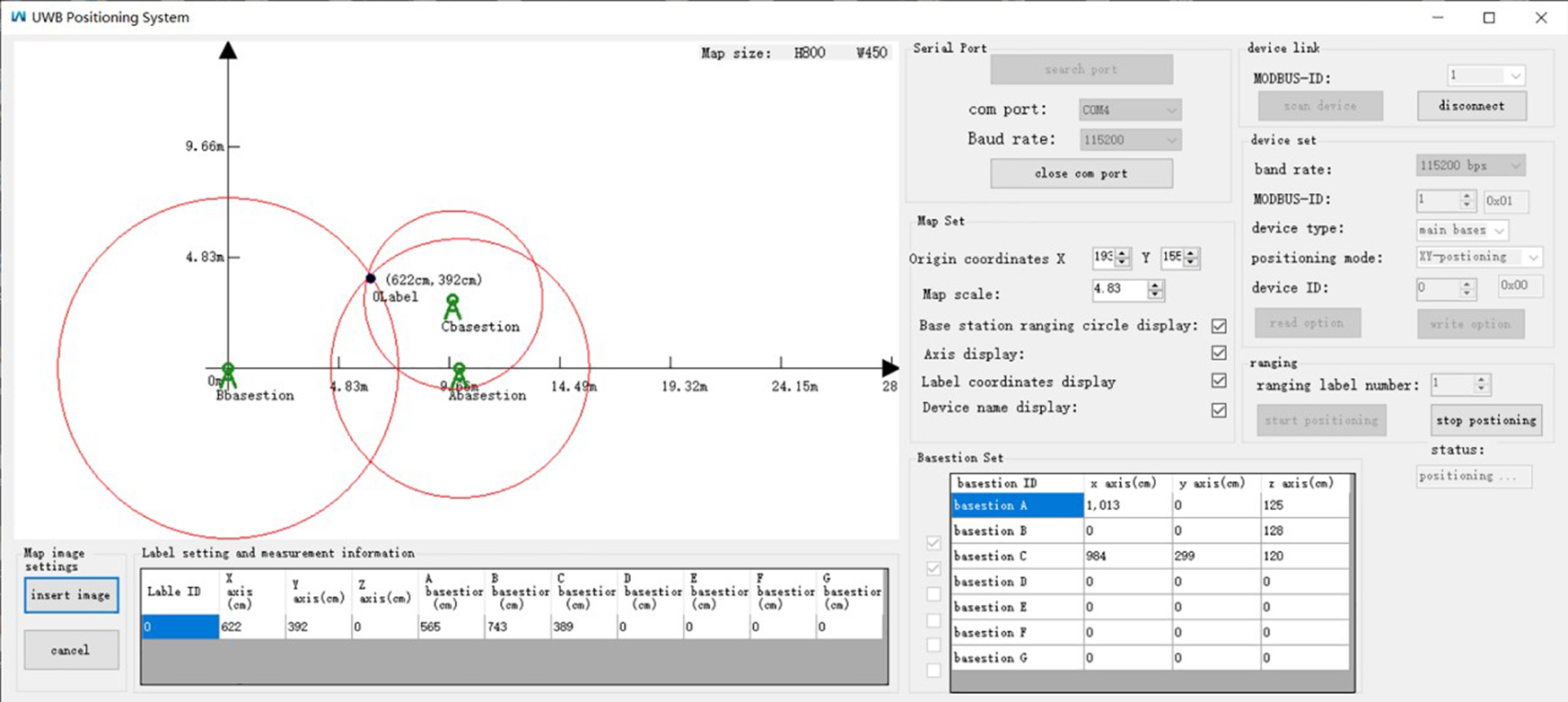

Supplement: Supplementary file 5 [file Image_5.jpg]
